# Measurement Properties of Smartphone Approaches to Assess Physical Activity in Healthy Young People: Systematic Review

**DOI:** 10.2196/39085

**Published:** 2022-10-21

**Authors:** Belinda Parmenter, Claire Burley, Courtney Stewart, Jesse Whife, Katrina Champion, Bridie Osman, Nicola Newton, Olivia Green, Annie B Wescott, Lauren A Gardner, Rachel Visontay, Louise Birrell, Zachary Bryant, Cath Chapman, David R Lubans, Matthew Sunderland, Tim Slade, Louise Thornton

**Affiliations:** 1 School of Health Sciences University of New South Wales Kensington Australia; 2 Centre for Healthy Brain Ageing, Discipline of Psychiatry & Mental Health University of New South Wales Sydney, NSW Australia; 3 National Drug Research Institute and enAble Institute Faculty of Health Sciences Curtin University Perth, WA Australia; 4 The Matilda Centre The University of Sydney Sydney, NSW Australia; 5 Galter Health Sciences Library & Learning Center Northwestern University Feinberg School of Medicine Chicago, IL United States; 6 Centre for Active Living and Learning College of Human and Social Futures University of Newcastle Callaghan, NSW Australia; 7 Hunter Medical Research Institute New Lambton Heights NSW Australia; 8 Faculty of Sport and Health Sciences University of Jyväskylä Jyväskylä Finland

**Keywords:** smartphone, mobile phone, mHealth, prevention, risk, physical activity, sedentary behavior, young people

## Abstract

**Background:**

Physical inactivity is a preventable risk factor for several chronic diseases and one of the driving forces behind the growing global burden of disease. Recent evidence has shown that interventions using mobile smartphone apps can promote a significant increase in physical activity (PA) levels. However, the accuracy and reliability of using apps is unknown.

**Objective:**

The aim of our review was to determine the accuracy and reliability of using mobile apps to measure PA levels in young people. We conducted a systematic review guided by PRISMA (Preferred Reporting Items for Systematic Reviews and Meta-Analyses).

**Methods:**

Studies published from 2007 to 2020 were sourced from 8 databases—Ovid MEDLINE, Embase (Elsevier), Cochrane Library (Wiley), PsychINFO (EBSCOhost), CINAHL (EBSCOhost), Web of Science (Clarivate), SPORTDiscus (EBSCOhost), and IEEE Xplore Digital Library database. Studies were conducted in young people aged 10-24 years and without chronic illnesses, who evaluated a mobile app’s ability to measure PA. Primary outcomes included validity, reliability, and responsiveness of the measurement approach. Duplicate screening was conducted for eligibility, data extraction, and assessing the risk of bias. Results were reported as a systematic review. The main physical activity measures evaluated for each study were the following: total PA time (min/day or min/week), total moderate to vigorous PA per week, daily step count, intensity measure (heart rate), and frequency measure (days per week).

**Results:**

Of the 149 identified studies, 5 met the inclusion criteria (322 participants, 176 female; mean age 14, SD 3 years). A total of 3 studies measured criterion validity and compared PA measured via apps against PA measured via an Actigraph accelerometer. The 2 studies that reported on construct validity identified a significant difference between self-reported PA and the objective measure. Only 1 of the 5 apps examined was available to the public, and although this app was highly accepted by young people, the app recorded PA to be significantly different to participants’ self-reported PA.

**Conclusions:**

Overall, few studies assess the reliability, validity, and responsiveness of mobile apps to measure PA in healthy young people, with studies typically only reporting on one measurement property. Of the 3 studies that measured validity, all concluded that mobile phones were acceptable and valid tools. More research is needed into the validity and reliability of smartphone apps to measure PA levels in this population as well as in populations with other characteristics, including other age groups and those with chronic diseases.

**Trial Registration:**

PROSPERO CRD42019122242; https://www.crd.york.ac.uk/prospero/display_record.php?RecordID=122242

## Introduction

Physical inactivity is a preventable risk factor for several chronic diseases and one of the driving forces behind the growing global burden of disease [1,2]. Physical inactivity and excessive sedentary behavior are increasing, especially in young people. A review of Australia’s health published in 2018 showed that 92% of young people aged 13-17 years did not meet the physical activity (PA) guidelines of 60 minutes of moderate to vigorous intensity PA each day [1]. Similar global trends have also been reported [2-4]. In addition, independent of PA, total sitting time and TV viewing time are also associated with greater risk for several major chronic disease outcomes [5]. However, data from Australia’s health 2018 indicate that only 20% of young people meet the sedentary screen-based behavior guideline [1]. Recent cross-sectional and large population-based cohort studies published by the authors of this paper [6-8] (sample sizes of 3826, 6640, and 231,048 participants) indicated that, on average, 85.9% and 77.7 % of Australian adolescents engage in too much recreational screen time and do not meet the PA guidelines, respectively [6-8].

Recent evidence has shown that interventions using mobile smartphone apps or activity trackers can promote a significant increase in PA levels [9]. Smartphone apps can track PA and enable continuous self-monitoring and feedback on PA through heart rate, step counts, and exercise type, duration, and intensity. Other benefits of using smartphones for PA tracking are high rates of smartphone ownership in young people, widespread use of smartphone tools to track PA, tools being less burdensome than traditional measures (eg, standalone step counter, heart rate monitor, or pen and paper), and ability to be used in remote locations.

If step counts and exercise duration and intensity are to be effectively used as reference values for achieving recommended PA levels, we need to ensure that the tools we are using to measure activity are accurate for the population in which we are measuring them. The Lancet PA Series Working Group’s [10] current recommendation for continued improvement in monitoring PA to help guide policies to increase activity levels further highlights the need for accuracy and reliability of PA monitors via smartphone technology, which has developed significantly over the past decade. Previous research has assessed the accuracy of PA data measured by smartphones to influence PA [11]. However, to our knowledge, there is no systematic review of the literature with respect to the validity and reliability of using smartphones to quantify PA levels.

The promotion of PA, and tools available to monitor it, has emerged as an important area of research that has drawn increasing interest from researchers and health professionals in a variety of fields. The growing availability of inexpensive parts and equipment has led to the development of mobile devices such as smartphones, providing platforms for new opportunities in health care and the promotion of PA. Accelerometers have emerged as the most useful and extensive tool to capture and assess human physical activities in a continuous, unobtrusive, and reliable manner, but they are expensive and not practical in all settings. Recent evidence [9] has shown that using apps or PA trackers is effective at promoting PA; however, if PA recommendations for optimal health are based on minutes per week and at least 60 minutes per day for young people, we need to ensure that these measures are accurate and reliable.

The primary aim of this review was to assess the accuracy and reliability of using smartphones to measure PA levels in young people aged 10-24 years. This sample was chosen to avoid issues regarding generalizability and to enable a clearer understanding on the reliability of these measures to be established first (ie, by focusing on healthy young people with less variability caused by ageing, comorbidities, and health history); it was also chosen because of behavioral differences in populations (eg, smartphone use in younger compared to older adults).

The specific objectives of this review were to (1) identify and describe the ways in which PA has been measured using smartphones in young people; (2) describe and critically evaluate the available evidence on the measurement properties and feasibility of these measurement processes; and (3) provide recommendations on the most suitable and effective ways of measuring PA.

## Methods

This review was conducted according to a published protocol [12] and in line with the 2020 PRISMA (Preferred Reporting Items for Systematic Review and Meta-Analysis) reporting guidelines [13]. This review forms part of a larger review that has been registered with the International Register of Systematic Reviews in PROSPERO (CRD42019122242) [12,14]. This larger review aimed to examine the measurement properties of smartphone approaches to assess 6 key health behaviors (ie, PA, sedentary activity, sleep, diet, alcohol use, and tobacco use, also known as the ‘big six’) that are recognized risk factors related to the development of chronic diseases [15]. However, studies identified for inclusion were heterogeneous, recruiting different populations and using different measurement methods across various health behaviors. Therefore, only those studies that examined PA, specifically in healthy young people, were included in this review. Findings regarding alcohol use, tobacco use, and diet are described in Thornton et al [12,14]. Methods for this review continued to follow those outlined in Thornton et al [14], with any differences in methods presented in the following sections. Only articles published between 2007 to 2022 were searched because smartphones with large touchscreens (ie, where users input directly using their finger) were not available before.

### Search Strategy and Selection Criteria

A research librarian searched 8 web-based databases—Ovid MEDLINE, Embase (Elsevier), Cochrane Library (Wiley), PsychINFO (EBSCOhost), CINAHL (EBSCOHost), Web of Science (Clarivate), SPORTDiscus (EBSCOhost), and IEEE Xplore Digital Library database—as per the search strategy published in previous studies [12,14], using particular search terms (Table S1 in Multimedia Appendix 1). For inclusion in the current review, studies were required to describe a smartphone-based approach to assess PA in healthy young people and to report on at least one measurement property of this approach identified in the Consensus-Based Standards for the Selection of Health Measurement Instruments (COSMIN) taxonomy of measurement properties [16]. The COSMIN checklist assesses the methodological quality of studies investigating the measurement properties of patient-reported outcome measures. Measurement properties assessed in this study include internal consistency, reliability, measurement error, content, construct, criterion validity, and responsiveness; definitions are outlined in Table 1 according to COSMIN. Healthy young people were defined according to the World Health Organization as persons aged between 10 and 24 years [17] with no known chronic conditions. Outcomes of interest included measurement effectiveness (ie, accuracy and reliability) of a smartphone to measure PA, reported as total PA time (min/day or min/week), total moderate to vigorous PA per week, daily step count, intensity measure (heart rate or rate of perceived exertion), or frequency measure (days per week).

**Figure 1:**
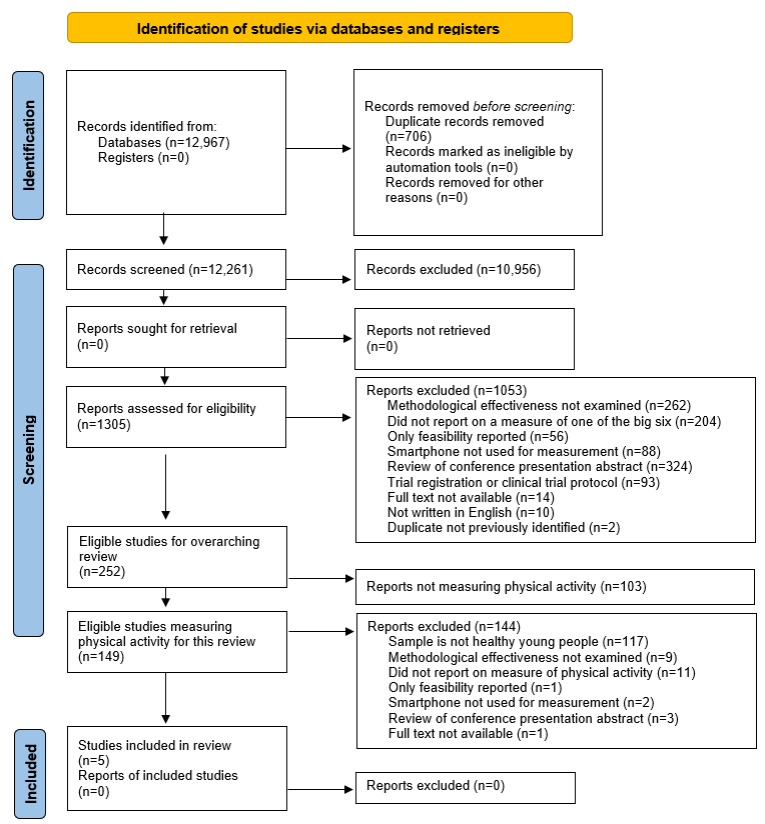
PRISMA (preferred reporting items for systematic review and meta-analysis) flow chart of the search strategy outcomes, including those of the larger review conducted by Thornton et al [12,14], followed by the narrowed search on studies measuring physical activity in healthy young people. Big six: 6 identified risk factors related to the development of chronic disease [15]: physical inactivity, sedentary time, poor sleep, poor diet, and alcohol and tobacco use.

Studies were excluded if participants were not healthy young adults; they were also excluded if they did not examine methodological effectiveness; did not report on PA and feasibility; did not use smartphones; were not published in English; if they described feasibility of the measurement approach only; described measurement properties of using text messaging only to measure behaviors; and described the measurement properties of a wearable device (eg, Fitbit) alone (Figure 1).

**Table 1:** Measurement validity criterion types and their definition according to Consensus-Based Standards for the Selection of Health Measurement Instruments (COSMIN) assessed in this study.

Criterion	Definition
Internal consistency	The degree of interrelatedness among devices
Reliability	The degree to which the measurement is free from measurement error
Measurement error	The difference between a measured quantity and its true value
Content validity	The degree to which the device being assessed is an adequate representation of the construct being measured
Construct validity	The degree to which the results are consistent with the hypothesis, based on the assumption that the device validly measures the construct to be measured
Criterion validity	The degree to which the results are adequate reflections of a ‘gold standard’
Responsiveness	The ability of a device to detect change over time in the construct being measured

### Data Extraction

All identified studies were exported into Endnote (version X9) for removal of duplicates. Records were then uploaded to Covidence Systematic Review software (Veritas Health Innovation) for screening. Authors participating in the screening, full-text review, and data extraction process attended training sessions, where multiple reviewers independently reviewed and discussed the same selection of articles to help ensure consistency across reviewers. As described in Thornton et al [12,14] for the larger review, titles and abstracts were screened by one reviewer (OG, RV, JW, CS, LT, BO, LB, LG, OG, ZB, KC, or BP) and then the full text of potentially relevant studies was independently assessed for eligibility by at least two members of this group.

Titles and abstracts of included studies were then screened according to the specific inclusion criteria of this review by one reviewer (BP). The full texts of potentially relevant studies were independently assessed for eligibility by 2 authors of the research team (CS and JW), with any disagreements resolved with the assistance of a third or fourth researcher (LT and BP). CS and JW independently extracted data in duplicate using a standardized extraction form to ensure that it adequately captured trial data, and they agreed on final reported data. Further details of data extraction are included in a previously published protocol [12].

### Physical Activity Measures, Measurement Validity, and Risk of Bias

The primary outcomes of interest for this review were PA measurement properties of smartphone-based approaches to assess PA. Specifically, we investigated, as reported in included studies, the reliability, measurement error, content validity, construct validity (including convergent validity, structural validity, and cross-cultural validity), criterion validity, and responsiveness of the identified approaches. In this review, for studies to be classified as measuring criterion validity, the smartphone-based approach of interest must have been compared to an objective measure of PA that had already been tested for reliability (eg, step count using a pedometer with an internal spring that moves up and down with hip motion or an Actigraph accelerometer that uses small motion sensors to measure acceleration along 3 axes) [18]. Where the smartphone-based approach was compared to a self-report measure, even if it was described as the gold standard method by the studies’ authors, the paper was classified as investigating construct and specifically convergent validity. For this review, the gold standard measurement tool for PA was classed as the Actigraph accelerometer [19,20]. Risk of bias of the included studies was assessed using the COSMIN risk of bias checklist [16,21,22].

## Results

### Summary of Included Studies

Of the 12,967 records identified through the search strategy (Figure 1), 149 studies met the PA smartphone criteria for this review without restricting studies by age. In summary, articles were excluded for not examining the methodological effectiveness of the measurement approach, not reporting on at least one of the ‘big six’ [15] or on feasibility, and not using a smartphone (Figure 1). When applying the inclusion criteria for participants to be healthy young people, only 5 studies [23-27] were eligible. The key characteristics of the included study populations are presented in Table 2. In total, 322 young people were included in the studies, and 176 were female, with a mean age of 14.3 (SD 3.2) years. Only 2 studies reported the age range of participants. Jongprasithporn et al [25] included participants aged 18-23 year, whereas Dunton et al [24] studied participants aged 9-13 years. As 90% of the participants were aged 10-14 years in the study by Dunton et al, the authors decided to include it in this review. Two studies reported the study was conducted with young people who were overweight or obese with a mean BMI of 32.7 kg/m^2^ [27] and 31.3 kg/m^2^, respectively [26].

Two studies were conducted in the United States [23,24], two in Germany [26,27], and one in Thailand [25]. Of the included 5 studies, all were assessing accuracy of smartphone apps collecting PA data, compared to the more subjective self-reporting or objective accelerometers.

The following 3 studies measured criterion validity, comparing phone data to objectively measured and automatically collected data: Bruening et al [23] completed an accuracy study to test the validity of a mobile ecological momentary assessment (EMA) methodology to assess PA levels ; Dunton et al [24] conducted a validity and feasibility study investigating electronic EMA of PA via smartphone-administered surveys; and the final study [25] examined the accuracy of the Thai 3 Axis (or TH3AX) smartphone app to assess PA. The 2 studies that measured construct validity [26,27] were pilot studies conducted on a group participating in an existing hospital treatment program for weight loss. Both studies assessed the feasibility and acceptability of modern electronic health care technology in treatment compared to self-report, where the user manually recorded bouts of PA across the study period.

Characteristics of included studies are outlined in Table 3. With respect to the apps used to record the PA data, 3 studies did not report the name of the app used, one used a mobile EMA app called devilSPARC [23], and one study [25] used the TH3AX app, which records PA data in real time. Four studies [23-25,27] did not report if the apps were available to the public. Schiel et al [26] reported the app was available to the public, but it could not be located in the Apple or Google Play app stores.

In 2 studies [23,24], participants were sent real time prompts throughout the day to answer questions about the activity they were undertaking. The 3 remaining studies [25-27] used real-time monitoring of PA though mobile phone motion. Only one of the studies [25] that used passive motion sensing reported where the participants were instructed to wear the phone; this study asked participants to attach the phone to their right hip in the first test and then the right anterior superior iliac spine in the second test. Of the 5 studies, 4 monitored activities over an average of 4 days. The remaining study [25] monitored activities over a range of performance trials, where participants randomly took part in standing, walking, and running activities. All 5 studies examined PA measurement on Android phones, with only 1 study [23] looking at the feasibility of PA measures on the Apple iOS. In this study, to those participants who were interested in participating but did not own an Android or iOS mobile phone, a Motorola Moto G was loaned to be used for the duration of the study. Only two [23,24] of the 5 studies mentioned the actual type and brand of the phones used. Of the 5 studies, 2 reported that they offered financial incentives (US $80 and US $40) for participating in the study [23,25].

Two studies [23,24] required the user to actively enter bouts of PA data. In the 3 remaining studies [25-27], PA data were automatically collected through the motion or movement of the mobile phone. Two studies [26,27] used a mobile motion sensor that was integrated within the phone. One study [25] used an accelerometer that was built into the phone. All 3 of these studies [25-27] reported the algorithms used to compute PA behavior; however, they did not report whether the algorithm used was accessible via open source. None of the studies reported if participants wore a PA tracking monitor for the duration of the study. Sedentary behavior was measured in 4 of the 5 studies, with 2 [26,27] recording it objectively and automatically through the phone, and 2 through subjective self-report measures [23,24].

**Table 2:** Characteristics of the included study populations.

Author and year	Population in the study						Instrument administration			
	Sample size (n)	Age (years), mean (SD)	Age (years), range	Gender (female), n (%)	Other characteristics	Setting		Country	Language	Device
Schiel (2010) [27]	30	14 (3)	NR^a^	14 (47)	Overweight or obese (BMI 32.7 kg/m^2^)	Hospital treatment program		Germany	English	Android smartphone
Schiel (2012) [26]	124	13.5 (2.8)	NR	69 (56)	Overweight or obese (BMI mean 31.3, SD 5.2 kg/m^2^)	Weight reduction program		Germany	English	Android smartphone
Dunton (2013) [24]	121	11 (NA^b^)	9-13	59 (49)	38% risk of overweight or overweight	4th-8th grade		United States	English	Android smartphone
Bruening (2016) [23]	41	18.7 (0.5)	NR	30 (73)	Friendship networks and weight-related behaviors	College freshman		United States	English	Android or iOS smartphone
Jongprasithporn (2017) [25]	6	NR	18-23	4 (67)	Normal weight (BMI 21.5 kg/m^2^)	Healthy young adults		Thailand	English	Android smartphone

^a^NR: not reported.

^b^Not available.

**Table 3:** Key characteristics of studies examining the measurement of physical activity via a smartphone.

Author and year	App name	Risk of bias properties assessed									
		Publicly available	Measurement approach	Internal consistency	Reliability	Measurement error	Content validity	Construct validity	Criterion validity	Responsiveness	Overall rating
Schiel (2010) [27]	NR^a^	NR	Passive^b^-objective	No	No	No	No	Yes	No	No	Inadequate
Schiel (2012) [26]	NR	Yes	Passive-objective	No	No	No	No	Yes	No	No	Inadequate
Dunton (2013) [24]	NR	NR	Active^c^ self-report	No	No	No	No	No	Yes	No	Very good
Bruening (2016) [23]	devilSPARC	NR	Active self-report	No	No	No	No	No	Yes	No	Very good
Jongprasithporn (2017) [25]	Thai 3 Axis	NR	Passive-objective	No	No	Yes	No	No	Yes	No	Very good

^a^NR: not reported.

^b^Passive: data automatically collected.

^c^Active: requires user to do something.

### Risk of Bias Measurement Properties

Overall, the use of measurement properties to assess reliability, validity, and responsiveness was poor, with each study only reporting on one measurement property completed. No studies looked at the reliability of their results through repeated measures. Only 1 study [25] reported on an overall measurement error, which was low at 0.12. No studies looked at structural validity, that is, the degree to which studies measured PA levels. No studies looked at cross-cultural validity and the applicability of results across other cultures.

Two studies reported on construct validity, both of which were conducted by the same research team [26,27]. In the first study [26], there were significant differences between self-reported PA and the PA measured by motion sensors in the phone. In general, the duration of PA documented by children and adolescents was much higher than the duration measured via motion sensors. Correlation analyses, however, revealed moderate to strong significant correlations between the calculated duration of PA and the time spent in activities such as cycling (*r*=0.67; *P*<.01), the calculated duration of PA and the total amount of activity units (*r*=0.89; *P*<.01), and the calculated duration of PA and energy expenditure (*r*=0.82; *P*<.01) [27]. This demonstrated the strong consistency between PA assessment via a mobile motion sensor board and reality [26]. In the second study [27], the duration of PA estimated by children and adolescents was also significantly higher compared to the measured values for walking and running in the first study [26]. There was no difference between the estimates for cycling in the two studies. There were also weak to moderate significant correlations between the total calculated duration of PA and the time spent in some of the different activities, such as cycling (*r*=0.67; *P*=.001), driving (*r*=0.46, *P*=.01). There was a strong significant correlation between the measured duration of PA and the total number of activity units (*r*=0.89, *P*=.001). There was also a strong significant correlation between the measured duration of PA and the estimated energy expenditure (*r*=0.82, *P*=.001) [27].

The 3 remaining studies [23-25] specifically investigated criterion validity, where the app was compared to a previously validated objective measurement [22]. Although, one study [24] reported it as construct validity. In Dunton et al [24], the methodology of EMA activity responses was tested by examining differences in the mean number of steps (measured by an accelerometer) across EMA-reported activity categories. We have, therefore, reported this under criterion validity. Across both weight status groups, steps were significantly higher for EMA surveys reporting active play, sports, or exercise compared to any other type of activity. In addition, the mean number of steps recorded while talking on the phone, doing chores, riding in a car, and something else were significantly greater than mean steps recorded while reading, using a computer, doing homework, watching TV or movies, and playing video games [24]. However, this study concluded that it is acceptable and valid to use mobile phone EMA technology to measure PA and sedentary behavior in children aged 9-13 years during leisure time. In the second study [23], the odds of a participant having their accelerometer-derived activity level match their reported PA level were significant for mobile-based EMA-reported sedentary PA, light PA, and moderate PA. Due to only one participant having vigorous accelerometer values, odds were not computed for vigorous activity. The match rates were highest for EMA-reported sedentary and light PA (340/565, 60.3% and 37/63, 58.7%, respectively) and lowest for moderate PA (9/40, 22.5%) and vigorous PA (1/26, 3.8%). This study concluded that the devilSPARC mobile EMA app is valid for assessing the presence of sedentary activities during the day [23]. The third study [25] on criterion validity reported that the lowest sensitivity (0.975) of the TH3AX app was computed during standing activity. The highest sensitivity (0.988), specificity, and accuracy were all identified during running activity. The average sensitivity, specificity, and accuracy of TH3AX for standing, walking, and running were 0.981, 0.988, and 0.986, respectively. Based on these results, the authors validated the use of the smartphone app for activity recognition in young people. No studies looked at the responsiveness of the apps used and how well they detected change over time in PA levels.

## Discussion

The primary aim of this review was to assess the accuracy and reliability of using smartphones to measure PA levels in young people aged 10-24 years. In summary, 5 studies met the inclusion criteria (including 322 young people, with a mean age of 14.3, SD 3.2 years) and objectively assessed the accuracy of smartphone apps to collect PA data. Data were either collected automatically via phone movement or manually by the user. The overall rating for 3 studies was considered ‘very good,’ and the remaining 2 studies were rated ‘Inadequate’ (Table 3). Only one study reported that the app was available to the public [26], but it could not be located in the Apple or Google Play app stores.

Results from this review suggest that much more research is needed on validating these apps against the gold standard tools such as the Actigraph accelerometer. With only 5 studies eligible for inclusion in this review, and only 3 of those studies comparing mobile apps to more traditional objective measures of PA, more research is needed on the efficacy and reliability of mobile phone tracking and monitoring of PA in young people. Furthermore, when reporting on the outcomes of these studies, more information should be provided on participant characteristics. Most of the studies in this review only reported on age and gender, with 3 of them providing information on weight status. Since only over 18% of children and adolescents in the world were reported as overweight or obese in 2016 [28], future studies on PA should start reporting weight status of young people to assist with identifying interventions that may reduce weight in this population.

It is crucial that studies report the most accurate placement site for general community use, as most people tend to carry their mobile phones in their pockets or handbags. For objective monitoring, the attachment site of the phone and the place of the accelerometer to which the phone was compared is important information for people relying on mobile phone data for PA monitoring. Previous work from our group showed that some motion senses and activity trackers work better and more accurate on specific parts of the body [29].

Of the 5 apps examined in this review, only 1 app is publicly available, and 3 of the 5 studies did not report on the public availability of the apps the researchers used. PA tracking apps should be publicly and freely available to have the greatest impact on improving PA levels and preventing the development of chronic diseases. In addition, with the Apple iPhone being the most popular smartphone on the market, more studies are needed on the efficacy of PA measurements using this system.

### Limitations

Due to the small number of studies examining the efficacy, reliability, and validity of mobile apps measuring and tracking PA we were unable to complete a meta-analysis on the data. Each study’s methodology was so different, and it was not practical to combine study data. Some studies did not report on the actual measurement property used to examine reliability, validity, or responsiveness. In this study, we analyzed the information provided by one group under criterion validity, even though it was reported in the study as construct. However, as the methods were so detailed, we were able to identify the correct measurement property used. The range of sample sizes across the 5 studies was large, from 6 to 124, and sample size calculations were not reported. In addition, the measurement approach of each study also ranged widely between using a passive-objective or active self-report approach.

### Conclusions

Few studies have examined the accuracy, validity, or reliability of smartphones measuring and tracking PA levels in healthy young people. Of the 3 studies that measured validity against an objective measure of PA, such as the Actigraph accelerometer, all concluded that mobile phones were acceptable and valid tools. However, more research is needed that focuses on population characteristics, such as gender, different age groups, disability, and chronic diseases. Establishing the validity and reliability of smartphones to measure PA levels in young people will allow further research to investigate their use to increase PA in this population (ie, prevention strategies for developing chronic diseases or treating them), as well as identifying suitability for use in other populations, such as older adults and people currently living with chronic conditions.

## Appendix

Multimedia Appendix 1 Table S1 in Multimedia Appendix 1.

## Abbreviations

EMA: ecological momentary assessment
PA: physical activity
PRISMA: preferred reporting items for systematic review and meta-analysis
